# Supplementary material for: Brazilin is a natural product inhibitor of the NLRP3 inflammasome
Source: iScience. 2024 Jan 19;27(2):108968. doi: 10.1016/j.isci.2024.108968 (PMC10847679; doi:10.1016/j.isci.2024.108968)
Supplement: Document S1. Figures S1–S4 and Table S1 [file mmc1.pdf]

iScience, Volume *27*

Supplemental information

Brazilin is a natural product inhibitor of the NLRP3 inflammasome

Emily McMahon, Sherihan El-Sayed, Jack Green, Christopher Hoyle, Lorna FitzPatrick, Emma V. Jones, Eve Corrie, Rebecca L. Kelly, Mairi Challinor, Sally Freeman, Richard A. Bryce, Catherine B. Lawrence, David Brough, and Paul R. Kasher

Supplemental Figures and Tables

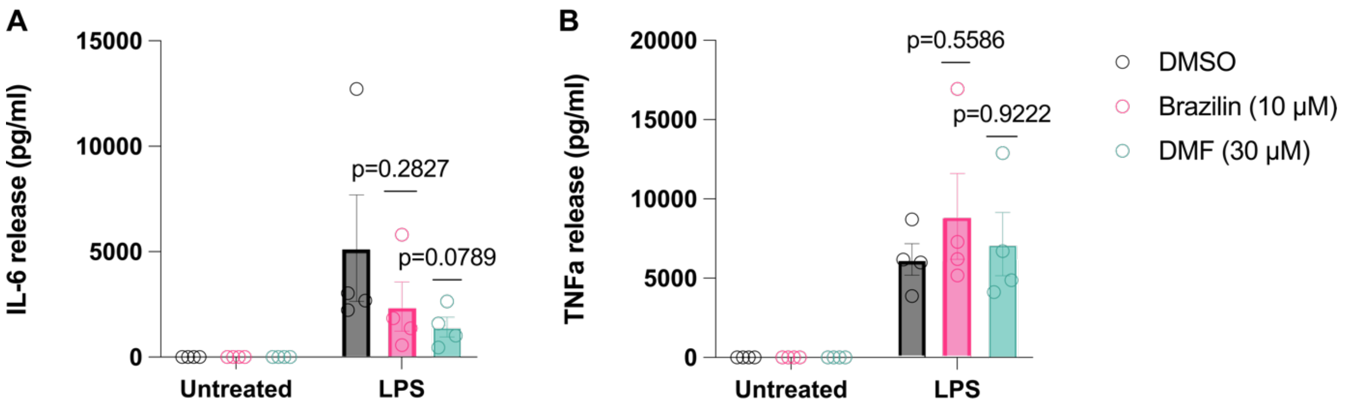

Figure S1 **Supernatant cytokine release from primary BMDMs after LPS priming**, related to Figure 3. BMDMs were treated with vehicle (DMSO), brazilin (10 µM) or DMF (30 µM) for 15 min. LPS (1 µg ml^{-1}, 6 h) was then added to the wells to induce priming. Supernatants were analysed by ELISA for **(A)** IL-6 (N=4) and **(B)** TNFα (N=4) release. Data show absolute cytokine release (pg ml^{-1}). Data are presented as mean ± SEM, each data point ('N') representing a biological repeat. Statistical analyses following normality testing: (A) Kruskal-Wallis test with Dunn's post hoc comparisons, LPS+ data; (B) one-way ANOVA with Tukey's post hoc comparisons, LPS+ data. *$p<0.05$. BMDMs, bone marrow-derived macrophages; DMSO, dimethylsulfoxide; DMF, dimethyl fumarate.

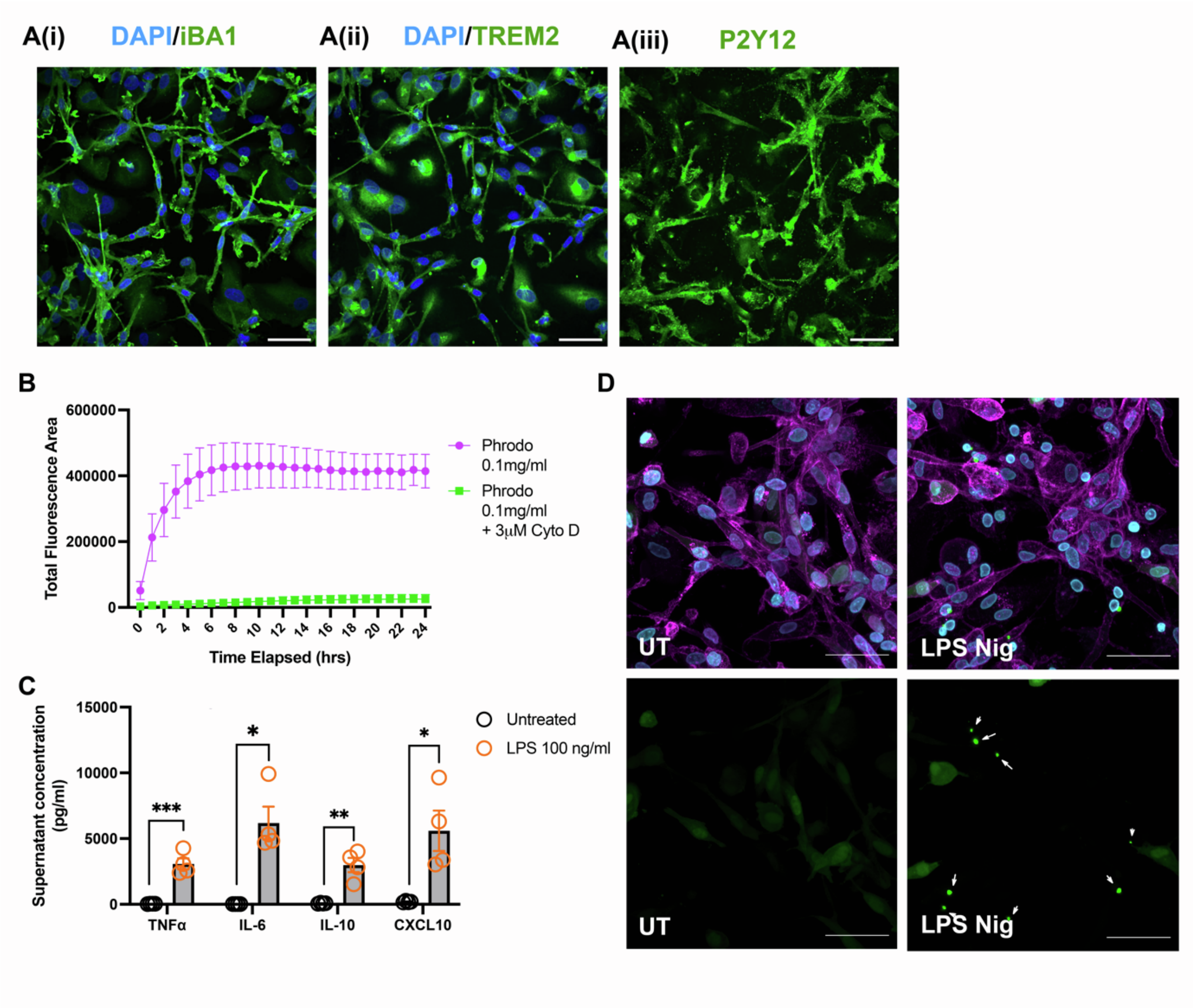

Figure S2 Characterisation of human iPSC-derived microglia demonstrates they are functional and respond to inflammatory stimuli, related to STAR methods.

(A) Representative fluorescent confocal microscopy images of iPSC-derived cells expressing markers typical of microglial cells: (Ai) iBA1, (Aii) TREM2 and (Aiii) P2Y12 (all green GFP). Nuclei stained with DAPI (blue). Scale bars represent 50 µM. **(B)** iPSC-microglia display the ability to phagocytose. Here shown is engulfment of pHrodo labelled E.coli bioparticles, which fluoresce once inside acidic phagosomes. Cytochalasin D (3µM) was added as a negative control to inhibit phagocytosis (n=4). **(C)** iPSC-microglia respond to LPS, releasing cytokines and chemokines. Measurement of supernatant cytokines/chemokines was performed using Luminex technology (n=4). **(D)** The formation of ASC specks can be seen in iPSC-derived cells transfected with lentiviral hASC-GFP. ASC specks are not present in untreated (UT) iPSC-microglia, but specks form in response to treatment with LPS (100 ng ml^{-1}, 16 h) followed by nigericin (10 µM, 2 h). Arrow heads point to ASC specks. Wheat Germ Agglutinin (WGA) cell mask (magenta) and Hoescht nuclear stain (blue) are also shown. Scale bars represent 50 µM. All images were acquired using an Opera Phenix fluorescent confocal microscope. Data are expressed as mean ± SEM. (C) Unpaired t-tests were used to compare TNFa, IL-10 and CXCL10 release between untreated and LPS treated cells. A Mann-Whitney test was used to compare IL-6 release between untreated and LPS treated cells. Each datum represents a biological repeat (‘n’).

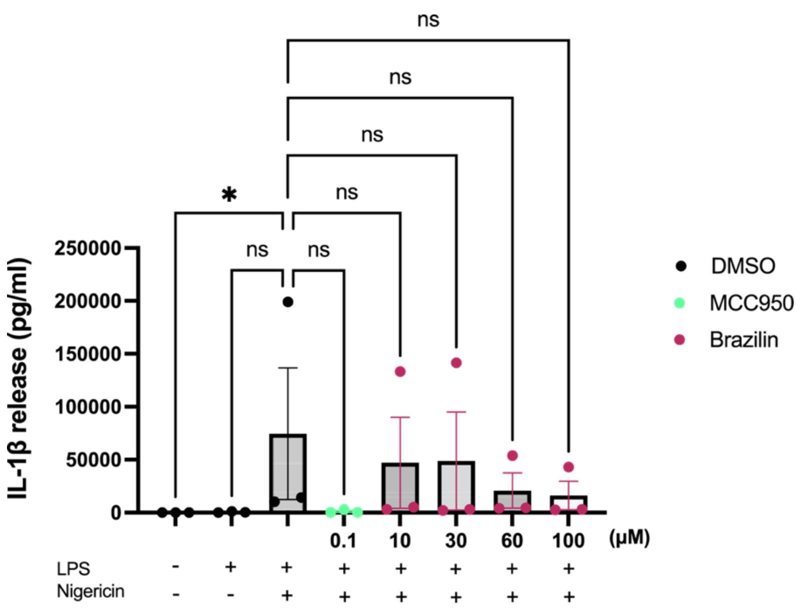

Figure S3 **Supernatant IL-1β released from human iPSC-microglia,** related to Figure 5.

Cells were primed with LPS (100 ng ml^{-1}) or vehicle (16 h) before treatment with MCC950 (0.1 µM), Brazilin (10, 30, 60 or 100 µM) or DMSO control (30 mins). Nigericin was then added to activate the NLRP3 inflammasome (10 µM, 2 h). Supernatant IL-1β was quantified using an IL-1β Human Luminex® Discovery Assay kit. Kruskal-Wallis test with Dunn's multiple comparisons. All data are expressed as mean ± SEM. *$p<0.05$. Each data point ('N') represents a biological repeat.

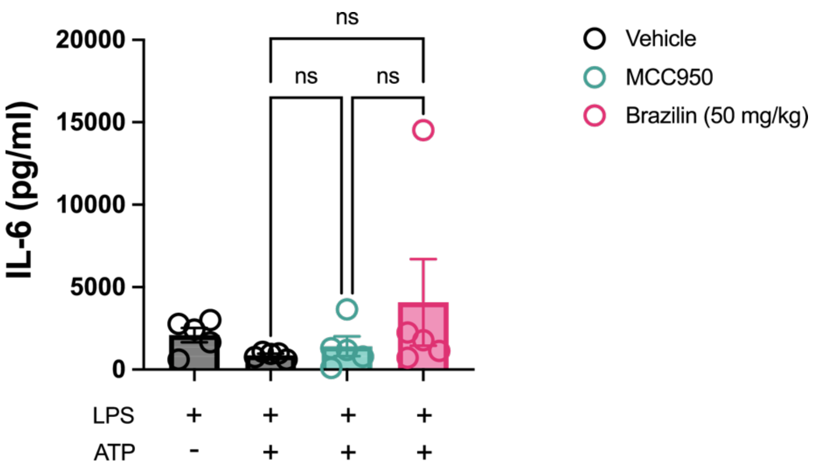

Figure S4 Pre-treatment with brazilin does not alter peritoneal IL-6 release in response to LPS and ATP administration, related to Figure 6.

Male C57 mice (N=5 mice/group) received a single i.p. dose of either vehicle (1% v/v DMSO in PBS), brazilin (50 mg/kg) or MCC950 (20 mg/kg), alongside LPS (1 µg; i.p.). After 4 hours, mice received a single i.p. dose of ATP (100mM in PBS) or vehicle (PBS), 15 minutes prior to peritoneal lavage. Data are presented as mean ± SEM. One-way ANOVA with Tukey's post-hoc comparisons, to assess the effect of drug treatment between groups treated with both LPS and ATP. DMSO, dimethylsulfoxide; LPS, lipopolysaccharide; ATP, adenosine triphosphate; I.P., intraperitoneal.

Table S1 **Average interatomic distances (in Å) between NACHT residues and selected carbon atoms of S-brazilin and S-brazilein from the 40 ns MD of ligand-NACHT complexes**, related to Figure 1, Table 1 and Video S1. The initial distance in docked poses was measured using MOE. Standard deviations in parentheses.

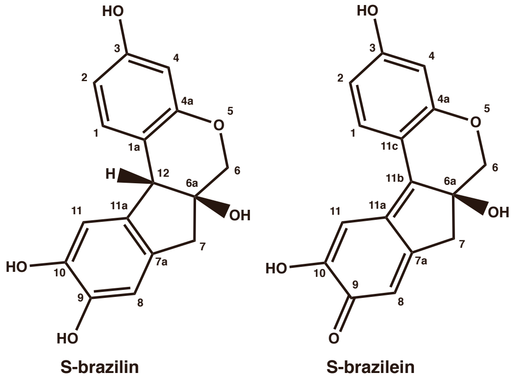

Distance	S-brazilin		S-brazilein	
	Initial	MD	Initial	MD
C2-Leu413C_{β}	3.85	4.08 (0.27)	3.60	5.04 (0.92)
C3-Leu413$C_{\delta 1}$	4.43	4.40 (0.44)	3.89	4.84 (1.00)
C4a-Ile234$C_{\delta 1}$	3.89	4.16 (0.43)	4.71	5.59 (1.13)
C7-Ile151$C_{\delta 1}$	5.28	4.26 (0.36)	4.59	4.76 (0.70)
C8-Tyr381$C_{\varepsilon 2}$	4.27	4.36 (0.36)	3.99	4.38 (0.41)
C9-Tyr168$C_{\delta 1}$	4.03	4.69 (0.32)	4.10	4.42 (0.39)
C10-Ile234$C_{\gamma 2}$	4.12	3.88 (0.29)	3.73	4.46 (0.44)
C11-Ile234$C_{\gamma 1}$	4.39	3.87 (0.30)	3.92	4.82 (0.59)
C11-Pro412C_{β}	3.09	3.58 (0.24)	3.64	4.22 (0.57)
